# Neuroprotective Activity of Sitagliptin via Reduction of Neuroinflammation beyond the Incretin Effect: Focus on Alzheimer's Disease

**DOI:** 10.1155/2018/6091014

**Published:** 2018-08-16

**Authors:** Michał Wiciński, Eryk Wódkiewicz, Maciej Słupski, Maciej Walczak, Maciej Socha, Bartosz Malinowski, Katarzyna Pawlak-Osińska

**Affiliations:** ^1^Department of Pharmacology and Therapeutics, Faculty of Medicine, Collegium Medicum in Bydgoszcz, Nicolaus Copernicus University, M. Curie 9, 85-090 Bydgoszcz, Poland; ^2^Department of Hepatobiliary and General Surgery, Faculty of Medicine, Collegium Medicum in Bydgoszcz, Nicolaus Copernicus University, M. Curie 9, 85-090 Bydgoszcz, Poland; ^3^Department of Obstetrics, Gynecology and Gynecological Oncology, Faculty of Medicine, Collegium Medicum in Bydgoszcz, Nicolaus Copernicus University, Ujejskiego 75, 85-168 Bydgoszcz, Poland; ^4^Department of Pathophysiology of Hearing and Balance System, Faculty of Medicine, Collegium Medicum in Bydgoszcz, Nicolaus Copernicus University, M. Curie 9, 85-090 Bydgoszcz, Poland

## Abstract

Sitagliptin is a member of a class of drugs that inhibit dipeptidyl peptidase (DPP-4). It increases the levels of the active form of incretins such as GLP-1 (glucagon-like peptide-1) or GIP (gastric inhibitory polypeptide) and by their means positively affects glucose metabolism. It is successfully applied in the treatment of diabetes mellitus type 2. The most recent scientific reports suggest beneficial effect of sitagliptin on diseases in which neuron damage occurs. Result of experimental studies may indicate a reducing influence of sitagliptin on inflammatory response within encephalon area. Sitagliptin decreased the levels of proinflammatory factors: TNF-*α* (tumor necrosis factor-*α*), IL-6 (interleukin-6), IL-17 (interleukin-17), and CD-163 (cluster of differentiation 163), and contributed to an increase in levels of anti-inflammatory factors: IL-10 (interleukin-10) and TGF-*β* (transforming growth factor *β*). Moreover, sitagliptin demonstrated antioxidative and antiapoptotic properties by modifying glutamate and glutathione levels within the region of hippocampus in mice. It has been observed that sitagliptin decreases accumulation of *β*-amyloid within encephalon structures in experimental models of Alzheimer's dementia. This effect may be connected with SDF-1*α* (stromal cell-derived factor 1*α*) concentration. Administration of sitagliptin caused a significant improvement in MMSE (Mini–Mental State Examination) tests used for assessment of dementias. The paper presents potential mechanisms of sitagliptin activity in conditions connected with neuroinflammation with special emphasis on Alzheimer's disease.

## 1. Introduction

The search for efficient neuroprotective drugs which would help in the treatment of acute and chronic brain diseases has lasted for years. Stroke is the cause of death of over 6 million people annually, which makes it the second most frequent cause of death worldwide. 1.5 million people die because of Alzheimer disease every year. These data imply a substantial need for application of new neuroprotective drugs [1].

Recently, there has appeared intriguing evidence based on experimental research and clinical trials indicating that new DPP-4 inhibitors (dipeptidyl peptidase 4),** sitagliptin** being a representative of which, have neuroprotective properties. These drugs are registered for diabetes mellitus type 2 treatment where their influence on active incretin levels is used. DPP-4 inhibitors are responsible for slowing down enzymatic degradation of GLP-1 (glucagon-like peptide-1). In consequence, these drugs prolong its half-life time in blood and enhance the effect of incretins' activity. GLP-1 increases food induced insulin secretion, decreases pancreatic glucagon secretion, and inhibits gastric emptying which slows the rate of absorption of nutrients into the blood. Therefore, GLP-1 may prolong the feeling of satiety and reduce appetite by direct impact on the lateral hypothalamus [2, 3]. Additionally, GLP-1 ensures *β*-cell insulin supplementation by increasing insulin gene transcription [4]. Studies conducted in mice suggest that GLP‐1 may enhance the expression of glucose transporters, glucokinase, K^+^_ATP_ channel subunits K_ir6.2_, and SUR1 which improves the capability of *β*‐cells to sense and respond to glucose [5, 6].

Sitagliptin ((*3R)-3-amino-1-[3-(trifluoromethyl)-6,8-dihydro-5H-[1,2,4]triazolo[4,3-a]pyrazin-7-yl]-4-(2,4,5-trifluorophenyl)butan-1-one*) is quickly absorbed after oral administration and its activity can be observed on average after 5 minutes. Its bioavailability at the dose of 100 mg equals 87% [7]. 90% inactivation of DPP-4 occurs as early as within 15 minutes following oral administration and is maintained on the level of about 80% for 24 h [8]. Food intake does not influence pharmacokinetics of the drug. It is mainly excreted unchanged by kidneys (80%) [9]. It does not inhibit nor induce cytochrome P-450 enzymes [10]. It is a substrate for cytochrome P-450 enzymes, CYP3A4 and CYP2C8, and also for P-glycoprotein. Its serum half-life time equals about 12.4 h [11, 12].

## 2. Anti-Inflammatory Activity

Inflammatory response constitutes a pathogenic component of numerous diseases connected with neuron damage [13, 14]. There is an extensive literature documenting that the brains and cerebrospinal fluid of AD patients contain a variety of proinflammatory mediators, including complement, cytokines and chemokines, acute phase proteins, and proteases, as well as reactive oxygen and nitrogen species that are responsible for the oxidative damage in the AD brain [15, 16] (reviewed in** Akiyama et al**.).

**Figure 1:**
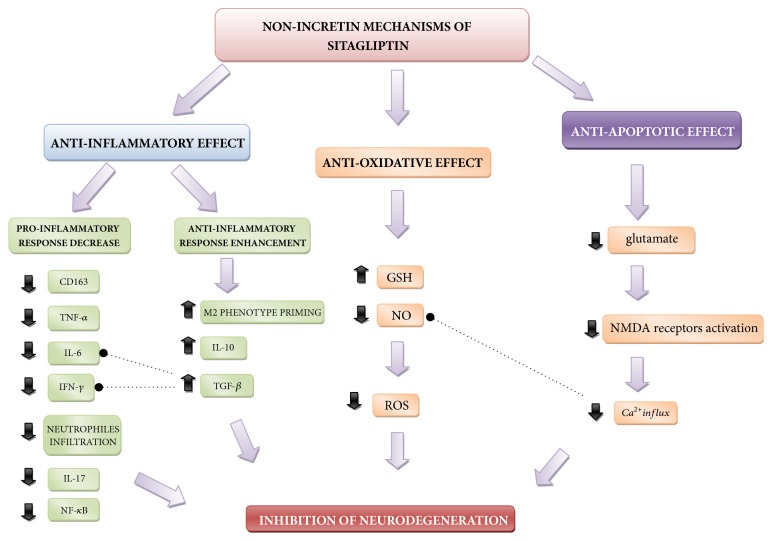
Proposed nonincretin mechanisms of sitagliptin activity.** CD163:** cluster of differentiation 163**, TNF-**α**:** tumor necrosis factor alfa,** IL-6:** interleukin 6,** IFN-**γ**:** interferon gamma,** IL-17:** interleukin 17,** IL-10:** interleukin 10,** NF-**κ**B;** nuclear factor kappa-light-chain-enhancer of activated B cells,** M2 phenotype priming:** priming of M2 macrophage phenotype,** TGF-**β**:** tumor growth factor beta,** GSH:** glutathione,** NO:** nitric oxide,** ROS:** reactive oxygen species, and** NMDA:** N-Methyl-D-Aspartate.

Newly discovered properties of sitagliptin allow for the consideration of the possibility of its use for reducing inflammatory reaction by interfering NF-*κ*B signaling cascade, among other proinflammatory pathways, and reducing excessive protein accumulation. The simplified diagram with possible mechanisms is presented in**Figure 1**.

An unexplained feature of normal aging is an increase in innate immune receptor expression in the brains of aging mice [17]. The enhancement of expression of these receptors occurs also in brains of AD patients [18, 19] and in animal models of the disease [18–20]. The AD mouse model was reported to have higher TLR4 (toll-like receptor 4) mRNA expression as compared to age matched controls. TLRs are a class of proteins that play a key role in the innate immune system. They are usually expressed on sentinel cells such as macrophages and dendritic cells that recognize structurally conserved molecules derived from pathogens [21]. Moreover, brains of AD mice exhibited pronounced TLR4 expression by immunofluorescence that was associated with A*β* plaque deposition in the entorhinal cortex [19, 20]. Interestingly, TLR4 polymorphism that exhibits a blunted TLR signaling response is associated with a 2.7-fold reduction in risk of late onset AD [22, 23]. Abovementioned reports arise the supposition that innate immune receptor expression may potentially play an important role in pathogenesis of AD.** Tang et al**. in their studies on mice neuronal cultures and human brain specimens postulated that the expression of TLR-4 is increased during exposure to A*β* (amyloid *β*) and HNE (4-hydroxy-2-nonenal) [24], the membrane lipid peroxidation product, created as a result of membrane-associated oxidative stress caused by the protein deposits. The number of TLR receptors increases in humans also as a result of stroke [25]. Higher expression of the receptors may have harmful implications. TLR4s activation initiates NF-*κ*B (nuclear factor-*κ*B) signaling pathway [26]. NF-*κ*B is a protein complex that controls transcription of DNA and plays a key role in regulating the immune response to infection as well as neuroinflammatory gene signaling events in AD brain. [16, 27, 28] They also revealed that JNK (jun N-terminal kinase) and caspase-3 activity levels were increased in neurons exposed to A*β* and HNE. Selective inhibition of TLR4 function showed the abilities of A*β* and HNE to activate JNK and caspase-3. Nevertheless, after the inhibition of TLR4 activity of JNK, caspase-3 was significantly suppressed. These findings suggest that neurons expressing TLR4 are vulnerable to degeneration in AD, by activating proapoptotic cascade involving JNK and AP-1. Consequently, a decrease in JNK and NF-*κ*B signaling pathway activation may have potential therapeutic effect [24].** Antoine Makdissi et al**. demonstrated for the first time in the human that sitagliptin exerts a comprehensive and potent anti-inflammatory and antiapoptotic effect. There was a suppression of intranuclear NF-*κ*B binding and the expression of IKK*β* (inhibitor of nuclear factor kappa-B kinase subunit beta), CCR-2 (C-C chemokine receptor type 2), TLR-2, and CD26 (cluster of differentiation-26 also known as DPP-4) in 2 hours after administration. This suppression was maintained for IKK*β*, CCR-2, TLR-2, and CD26 after 12 weeks. In addition, there was a suppression of the expression of TLR-4, JNK-1 (jun N-terminal kinase-1), and TNF*α* after 12 weeks [29]. TLR-4 activation initiates proapoptotic signaling cascade which involves JNK and AP-1 (activator protein-1) [30].

**El-Sahar et al**. in their studies demonstrated a decrease in markers of neutrophil granulocyte influx, i.e., MPO3 (myeloperoxidase-3, which is a lysosomal protein stored in azurophilic granules of the neutrophil and released into the extracellular space during degranulation) and inflammatory markers such as TNF-*α*, IL-6, or NF-*κ*B (also confirmed in other studies in mice [31]) in the hippocampus of rats receiving sitagliptin for 2 weeks prior to the induction of ischemia. Premedication with sitagliptin caused an increase of IL-10 level (which is known for its anti-inflammatory activity), both in rats with DM2 and in those not suffering from diabetes [32]. Congruous results were obtained in other studies in mice, additionally achieving analgetic effect by decreasing skin tenderness in pharmacologically induced arthritis. This activity might have resulted from limiting inflammatory reaction [33, 34].

A similar effect was observed by** Satoh-Asahara et al**. in people. In the course of studies in DM2 patients, sitagliptin caused a decrease in inflammatory markers, i.e., CRP, TNF-*α*, as well as a substantial augmentation in GLP-1 and IL-10 plasma concentration. The abovementioned changes were not observed in the group not treated with sitagliptin. A less spectacular, yet noticeable tendency was observed for lowering plasmatic and monocytic IL-6 levels [35, 36].

An immune response of microglia may have its role in pathophysiology of neurodegenerative diseases.** Bonaiuto et al**. indicated *β*-amyloid participation in the induction of proinflammatory cytokines by microglia in cell lines. *β*-amyloid molecules at suboptimal concentrations cause NF-*κ*B factor activation in the presence of IFN-*γ*, whereas at higher concentrations they are able to activate it even without interferon participation [31, 37, 38].

Sitagliptin appears to have an influence on inflammatory reaction with the participation of macrophages (which are part of microglia). If it is demonstrated to have a significant effect in constraining disadvantageous outcomes of microglia activation, it may indicate its potential protective activity in Alzheimer's disease and also after stroke [33, 39–41].

In the course of postmortem human studies, it demonstrated a decreasing effect on the concentration of CD163 macrophage activation marker, which was independent of glucose metabolism. Enhanced macrophage activity and their phenotype conversion towards the M1 phenotype are associated with chronic inflammation [42]. The recent study on humans compared sitagliptin activity with an oral agent of hypoglycemic activity from the group of alpha glycosidase inhibitors (glimepiride). Sitagliptin decreased the level of CD163 in 37 patients with diabetes. Alpha glycosidase inhibitor failed to demonstrate such activity. No differences were observed in HbA1C and BMI between the groups [43]. These reports may suggest that sitagliptin possess possible anti-inflammatory activity which surpasses incretin effect.

Interestingly,** Brenner et al**. in their studies noticed a connection between the increase in the number of macrophages in the M2 phenotype in vessel walls and the lesions of arteriosclerotic character [41]. Also,** Xiong et al**. in their work stated that IL-10 and IL-4 (IL-10 increase was observed with administration of sitagliptin) [39] cause differentiation of microglia and macrophages into the M2 phenotype. They ascribe the following features to this phenotype: neuroprotective activity consisting in phagocytosis of cell remains, promoting neurogenesis, and supporting cell repair after cerebral ischemia [14].

In 2015, in long-term studies on mice models being on high-cholesterol diet, it was shown that sitagliptin substantially reduced sclerotic plaque build upon the aorta's walls. It was connected with a bigger proportion of macrophages of M2 phenotype. Moreover, SDF-1 chemokine receptor blocker caused substantial decrease in therapeutic effect [41]. Therefore, it can be assumed that the abovementioned sitagliptin effect on macrophages could be connected with this signaling pathway.

Last but not least, sitagliptin demonstrated immunosuppressive activity towards Th1/Th17 lymphocyte cell lines. Increasing concentration of TGF *β*-1 caused a reduction in the number of circulating CD4+ Th17 and T_reg_ lymphocytes and a diminution in the concentration of IFN-*γ* and IL-6 resulting from decreasing the number of CD4+/IFN-*γ*+ and CD4+/IL-17+ cells [44].

## 3. Antioxidative and Antiapoptotic Effect

It is known that oxidative stress and a cellular death as its consequence represent one of the main factors standing behind AD progression [45]. Accumulated *β*-amyloid may reduce cellular respiration processes in neuron and astrocyte mitochondria by inhibition of complexes I and IV. Disorders in reactions taking place in an ETC (electron transport chain) cause a sudden increase in the production of free radicals. The created ROS are capable of changing mitochondrial permeability transition pore (MPTP) which leads to death of cells containing presenilins. Gene mutation for presenilin 1 was linked to autosomal dominant AD [46–48]. It is logical to hypothesize that a fall in ROS may cause a therapeutic effect.

In mice studies of** El Sahar et al**. it was demonstrated that sitagliptin administration reduced the levels of glutamate and nitric oxide and increased glutathione concentration within hippocampus structures. Higher concentration of glutamate may have proapoptotic effect connected with NMDA receptor activation [33, 49–51]. Activation of these receptors leads to excessive influx of calcium ions, which in consequence causes intensified synthesis of nitric oxide and related peroxidation of lipid membranes of neurons** (Figure 1)**. The abovementioned processes enhance oxidative stress effect in the form of neuronal cell damage and their entrance into programmed cell death pathways. Glutathione, however, as basic nonenzymatic cellular antioxidant plays an extremely significant role in the process of disposal of free radicals of potential neurodegenerative effect [52–55].

## 4. Impact on *β*-Amyloid and NFTs Accumulation

On the histopathological level, Alzheimer's disease is connected with accumulation of extra- and intracellular residues called plaques—built from *β*-amyloid and NFTs (neurofibrillary tangles) —being aggregates of tau protein which binds to microtubules [56]. This process leads to massive degeneration and subsequently to death of neurons [57]. Undoubtedly, disappointment with the effects of therapeutic solutions imposes a search for new drugs which might influence deposition of pathological inclusions. Research conducted by** D'Amico et al. **evaluating the use of sitagliptin in AD mice indicated increased concentration of GLP-1 in the encephalon area and a noticeable (approximately 60%) decrease of *β*APP and A*β* residue accumulation within hippocampus area of mice after administration of sitagliptin in comparison with mice not treated with the drug. Moreover, a noticeable decrease in inflammatory markers expression and nitrooxidative stress was observed in the areas in which accumulation of proteins was limited. Mice treated with exendin, a glucagon-like protein-1 (GLP-1) receptor agonist, did not demonstrate a reduction in residues. Unfortunately, no positive behavioral changes were obtained in this study or the results concerning the use of sitagliptin were ambiguous in this respect [58].

Interestingly, the abovementioned SDF-1 may have its role in accumulating residues associated with AD. It was noticed in mice model that the SDF-1*α* subtype is connected with the inhibition of *β*-amyloid accumulation. SDF-1 is a substrate of DPP-4 which is the target of sitagliptin's activity. Inhibition of enzymatic decomposition of this cytokine by sitagliptin may constitute a potential mechanism of activity of this drug [59].

Attempts were made at efficiency verification of incretin drugs in NFTs accumulation. In two studies, a contrary influence of two different DPP4 inhibitors on tau protein phosphorylation was demonstrated.** Kim et al., 2012** [60] presented results in rats suggesting not only lack of therapeutic effect of sitagliptin in this respect, but also escalation of disorders. On the other hand,** Kosaraju et al., 2013** [61] using another drug from the same group in mice—saxagliptin—obtained a certain therapeutic effect. It might have resulted both from the difference in the manner of disorder induction in laboratory mice and from the difference in nonincretin activity of saxagliptin and sitagliptin. Further research is indispensable in order to verify validity of the abovementioned hypotheses.

## 5. Incretin Effect and Neurodegeneration

Persistent hyperglycemia and decrease in insulin sensitivity in CNS lead to neurodegeneration in several overlapping mechanisms including oxidative stress, mitochondrial dysfunction [62], and neuroinflammation [63, 64] that are observed in these disorders. Furthermore, chronic hyperglycemia generates AGEs (advanced glycation end products) and their RAGE receptor which provide critical links between diabetes and AD [65]. An increase in levels of inflammatory markers and oxidative stress resulting from type 2 diabetes were associated with neuronal cell damage in Alzheimer's disease.

Insulin resistance diminishes Akt protein activation which is responsible among others for inhibition of GSK3*β*-kinase. It triggers tau protein phosphorylation [65–67] and the subsequent accumulation of NFTs. Another effect of insulin resistance is the prolonged state of hyperinsulinemia and in consequence the sequestration of IDE (insulin degrading enzyme). IDE apart from the function of enzymatic insulin decomposition also degrades *β*-amyloid. Due to decreased concentration of IDE, the amounts of decomposed *β*-amyloid may also be limited [65, 68, 69].

In the case of lack of poor pharmacological control of DM2, high glucose concentrations in blood cause a glycation of numerous proteins and lipids transforming them into AGEs which are molecules associated with *β*-amyloid occurrence [70]. In their research in human,** Valente et al.** [71] indicated that particularly high concentrations of RAGE (receptors for AGEs) appear in brains of people simultaneously suffering from AD and diabetes, which suggests destruction of nerve cells by RAGE-dependent mechanisms.

Hyperglycemia also leads to production of free radicals [72]. Observations were made that oxidative stress may elevate *β*-amyloid production by increasing *β*-secretase and *γ*-secretase activity [73]. The aggregates created become the cause of inflammatory reaction. This is another illustration of the complexity and interdependence of pathomechanisms presented [74–76].

Recent studies indicate a common effect of cognitive function attenuation in the course of diabetes associated with abdominal obesity (which is very often connected with DM2) [77]. Several times throughout the follow-up studies by** Singh-Manoux et al.** and** Janghorbani et al.** or review articles by** Kiliaan et al. **and** Arnoldussen et al.** a connection between obesity and development of senile dementia has been pointed [78–81]. It was demonstrated that free fatty acids stimulate the assembly of both amyloid and tau protein in vitro [82]. As a result of obesity, the amount of FFA (free fatty acids) in blood increases, which causes low-grade inflammation [83] and its long-term persistence may be connected with the loss of neurons [81]. Following esterification reaction, fatty acids may pass across the blood-brain barrier [82] and activate TLRs-4 [84]. As mentioned before, the activation of this protein leads to the production of proinflammatory cytokines in astrocytes [85, 86]. Incretin effect of sitagliptin is connected with limiting the factors underlying the abovementioned pathomechanisms. Sitagliptin treatment corrected the glycaemic dysmetabolism, hypertriglyceridemia, and inflammation in ZDF rats [87]. It allows for consideration of a common ground for the neuroprotective activity of sitagliptin of immunological character, as well as the indirect role associated with maintaining appropriate glycaemia. On the other hand, some aspects remain inconsistent. The diabetic animals under sitagliptin therapy showed elevated TNF-*α* level at the end of the study conducted by** Ferreira et al.** [87]. Therefore, still a lot of uncertainty remains.

## 6. Influence on Cognitive Functions

Apart from changes in laboratory markers, the treatment with sitagliptin caused an improvement of cognitive functions in elderly people in both groups with and without AD. Studies were conducted in patients receiving antidiabetic drugs, i.e., sitagliptin, metformin, and insulin, in various combinations. Both insulin and sitagliptin demonstrated a positive effect on cognitive functions. Among 205 subjects, 17 received only sitagliptin and 11 only metformin. Sitagliptin administration caused a significant improvement in MMSE tests used for assessment of dementias. Metformin failed to yield similar results [88].

**Gault et al.** also obtained positive results in mice. He achieved a 20% improvement in memory tests after 21 days of sitagliptin administration on high-fat diet. In the same studies, the author presented evidence for a possible sitagliptin influence on neurogenesis, demonstrating increased deposits of DCX (doublecortin)—neuronal renewal marker—in the hippocampus areas in mice receiving sitagliptin [89, 90].

## 7. Summary

The information presented in**Table 1** allows for considering sitagliptin as a promising drug in the treatment of conditions other than type 2 diabetes. If DPP-4 inhibitors are demonstrated to have clinically meaningful antisclerotic activity in humans, one potential application may be to reduce the burden of certain neurodegenerative disorders. The moderation of free radicals creation and aggregation of *β*-amyloid may prove to be helpful in reducing neurodegeneration in the one connected with AD and stroke as well as the one resulting from chronic hyperglycemia of CNS. Potential properties stimulating neuronal renewal, if proven, would find application in the treatment of various forms of dementia. Additional studies are essential to verify efficacy of sitagliptin in conditions specified in the paper.

## Figures and Tables

**Table 1:** Summary of reviewed results. **↓**: reduction, **↑**: increase, **CRP: **C reactive protein, ***CD163:****cluster of differentiation 163 ****, TNF-***α***:****tumor necrosis factor alfa ****, IL-6:****interleukin 6 ****, IFN-***γ***:**** interferon gamma ****, IL-17:****interleukin 17 ****, IL-10:****interleukin 10, **** NF-***κ***B:****nuclear factor kappa-light-chain-enhancer of activated B cells ****, M2 phenotype priming:****priming of M2 macrophage phenotype ****, TGF-***β***:**** tumor growth factor beta ****, GSH:**** glutathione ****, NOx:**** nitric oxide ***, *β*APP**: beta amyloid precursor protein, **A**β**: **beta amyloid**, DCX+cells: **cells expressing doublecortin, **CD**_**4**_**+ Th17: **CD4 positive T helper17 cells producing interleukin 17, **IKK**β**:** inhibitor of nuclear factor kappa-B kinase subunit beta, **JNK-1: **jun N-terminal kinase-1, **MMSE,** Mini–Mental State Examination.

**Authors**	**Subject of study**	**Result**
**Satoh-Asahara et al. 2013**	Humans with DM2	↑IL-10, ↓CRP, ↓TNF-*α*, ↓IL-6, M2 type monocyte priming
**Hattori et al. 2017**	Humans with DM2	↓CD163 in serum
**Matsubara et al. 2013**	Humans with DM2 and CAD	↓CRP, improvement of endothelial function
**Makdissi et al. 2012**	Humans with DM2	↓IKK*β*, ↓JNK-1, ↓TNF-*α*, ↓NF-*κ*B
**Isik et al. 2017**	Humans with/without AD	Significant improvement in MMSE
**El-Sahar et al. 2015**	Wistar rats	↓NF-*κ*B, ↓TNF-*α*, ↓IL-6, ↑IL-10, ↓caspase-3, ↓NO_x_
**Nader et al. 2018**	Albino mice	↓NF-*κ*B, ↑GSH
**Brenner et al. 2015**	Charles River mice	M2 type monocyte priming, reduction of atherosclerotic plaques in aortic wall
**D'amico et al. 2010**	AD mice model	↓*β*APP and ↓A*β* deposition in hippocampus
**Gault et al. 2015**	NIH/OlaHsd high-fat fed mice	20% improvement in memory test, ↑DCX+cells
**Ferreira et al. 2010**	ZDF rats	↓CRP, ↑TNF-*α*
**Kim et al. 2012**	LETO and OLETF rats	↑tau protein phosphorylation
**Pinheiro et al. 2017**	Human peripheral blood cells	↑TGF-*β*, ↓CD_4_+ Th17, ↓IFN-*γ*, ↓IL-6
